# The p38α MAPK Function in Osteoprecursors Is Required for Bone Formation and Bone Homeostasis in Adult Mice

**DOI:** 10.1371/journal.pone.0102032

**Published:** 2014-07-09

**Authors:** Edgardo Rodríguez-Carballo, Beatriz Gámez, Lara Sedó-Cabezón, Manuela Sánchez-Feutrie, Antonio Zorzano, Cristina Manzanares-Céspedes, José Luis Rosa, Francesc Ventura

**Affiliations:** 1 Departament de Ciències Fisiològiques II, Universitat de Barcelona, IDIBELL, L'Hospitalet de Llobregat, Spain; 2 Departament de Patologia i Terapèutica Experimental, Universitat de Barcelona, IDIBELL, L'Hospitalet de Llobregat, Spain; 3 Institute for Research in Biomedicine (IRB Barcelona), Barcelona, Spain; 4 Departament de Bioquímica i Biologia Molecular, Facultat de Biologia, Universitat de Barcelona, Barcelona, Spain; 5 CIBER de Diabetes y Enfermedades Metabólicas Asociadas (CIBERDEM), Instituto de Salud Carlos III, Barcelona, Spain; Cincinnati Children's Hospital Medical Center, United States of America

## Abstract

**Background:**

p38 MAPK activity plays an important role in several steps of the osteoblast lineage progression through activation of osteoblast-specific transcription factors and it is also essential for the acquisition of the osteoblast phenotype in early development. Although reports indicate p38 signalling plays a role in early skeletal development, its specific contributions to adult bone remodelling are still to be clarified.

**Methodology/Principal Findings:**

We evaluated osteoblast-specific deletion of p38α to determine its significance in early skeletogenesis, as well as for bone homeostasis in adult skeleton. Early p38α deletion resulted in defective intramembranous and endochondral ossification in both calvaria and long bones. Mutant mice showed reduction of trabecular bone volume in distal femurs, associated with low trabecular thickness. In addition, knockout mice also displayed decreased femoral cortical bone volume and thickness. Deletion of p38α did not affect osteoclast function. Yet it impaired osteoblastogenesis and osteoblast maturation and activity through decreased expression of osteoblast-specific transcription factors and their targets. Furthermore, the inducible Cre system allowed us to control the onset of p38α disruption after birth by removal of doxycycline. Deletion of p38α at three or eight weeks postnatally led to significantly lower trabecular and cortical bone volume after 6 or 12 months.

**Conclusions:**

Our data demonstrates that, in addition to early skeletogenesis, p38α is essential for osteoblasts to maintain their function in mineralized adult bone, as bone anabolism should be sustained throughout life. Moreover, our data also emphasizes that clinical development of p38 inhibitors should take into account their potential bone effects.

## Introduction

During development, ossification depends on the activity of osteoblasts that are derived from mesenchymal stem cells. Throughout this process of osteoblastic differentiation, osteochondroprogenitors proliferate and go through a series of steps before becoming mature osteoblasts [1], [2], [3]. Furthermore, osteocytes are derived from terminally differentiated osteoblasts that remain embedded in the bone-mineralized matrix. Later on in adulthood, bone formation and remodeling remain very dynamic processes that rely on a tight balance between osteoclast resorption and new bone formation by osteoblasts. Any disparity between these two activities causes pathological states such as osteoporosis [4].

Many extracellular stimuli, such as mechanical stress, inflammatory cytokines and growth factors, have been described as regulators of osteoblast differentiation through p38 MAPK signalling [5]. In mammalian cells, four isoforms of p38 Mitogen-Activated Protein Kinases (MAPKs) have been described: p38α (MAPK14), β (MAPK11), γ (MAPK12) and δ (MAPK13) [6]. Some differences in activation have been shown between distinct isoforms, with p38α MAPK being one of the most abundant isoform in osteoblasts and bone [7]. p38 MAPKs are activated by MKK3 and MKK6, which are also downstream of several MAPKKKs, including TAK1, ASK1 and MLKs [6].

p38 MAPK activity, known to play an important role in several steps of the osteoblast lineage progression, is necessary but not sufficient for BMP-induced acquisition of the osteoblast phenotype [8], [9], [10]. Evaluation of these effects is often based on the commonly used inhibitor, SB203580, which only inhibits p38α and p38β isoforms. Biochemical analysis has identified key osteogenic genes whose expression and/or function are regulated by p38. Evidence shows that p38 activity is required for BMP-induced *Osx* expression in calvaria, as well as bone-marrow-derived mesenchymal stem cells [11], [12], [13]. Moreover, several reports indicate that p38 phosphorylates critical transcription factors involved in osteoblastogenesis such as DLX5, RUNX2 and OSX [7], [13], [14], [15], [16]. Phosphorylation by p38 regulates their transcriptional activity by promoting association with transcriptional coactivators and chromatin remodeling complexes [7], [13], [14], [17].

p38 signalling in early bone development has also been studied in mouse models. Analyses of mice lacking TAK1, MKK3 or MKK6 display profound defects in bone formation and development. However, these defects differ depending on anatomical location. For instance, only MKK6 contributes to calvarial mineralization [5], [7]. The study of developing long bones of mice with specific deletion of p38α in osteoblasts showed a progressive decrease in bone mineral density in cortical and trabecular bone [18]. Although existing reports indicate the role of p38 signalling in early bone formation and skeletogenesis, its specific contributions to adult bone remodelling are still to be clarified. In earlier models p38 signalling was impaired in osteochondroprogenitors or osteoblasts during early bone formation both in utero and perinatally [7], [18]. Furthermore, it has been hypothesized that, whereas p38α is required for early osteoblast differentiation, p38β is the main isoform involved in late maturation and postnatal function [7].

Here, we evaluated osteoblast-specific deletion of p38α to determine whether it is necessary in early skeletogenesis as well as for bone homeostasis in adult bones. Early p38α deletion results in defective intramembranous and endochondral ossification through decreased expression and function of osteoblast-specific transcription factors and their targets. More importantly, deletion of p38α at three or eight weeks postnatally leads to significantly lower trabecular bone volume at 30 weeks and lower cortical volume and thickness at 60 weeks. These results demonstrate that p38α plays an essential role in the maintenance of osteoblast function during bone remodeling and that clinical development of p38 inhibitors should take into account their potential effect on bone.

## Results

### Osteochondroprogenitor-specific deletion of p38α in mice

To determine the function of p38α MAPK, we generated mice whose p38α was selectively disrupted in osteochondroprecursors under the control of a tetracycline-responsive promoter (*Osx1-GFP::Cre*) [19]. *Osx1-GFP::Cre:p38a^flox/flox^* mice, grown in the absence of doxycycline treatment (hereafter referred to as p38α knockout mice, KO), were born with the expected Mendelian frequency. Their viability was indistinguishable from those of control mice (*p38a^flox/flox^*; FF).

**Figure 1:**
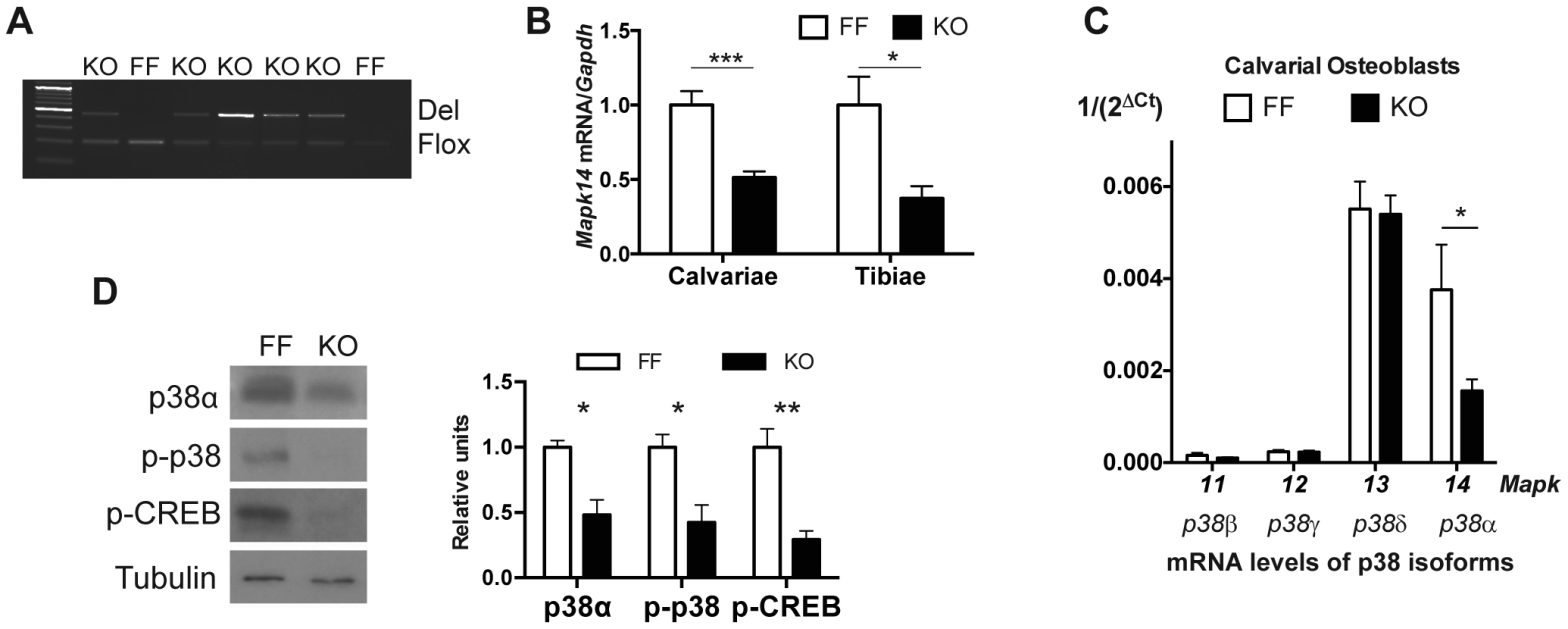
p38α specific-deletion in osteoblast progenitors. (A) PCR of calvarial bones of control (FF) and knockout (KO) mice using specific primers against *Mapk14* (*p38a*) demonstrate gene deletion in KO mice after Cre-mediated recombination. (B) qRT-PCR of mRNA from calvaria or tibia displays *p38α* (MAPK14) reduced expression in knockout versus control littermates (n = 7 for KO and 13 for FF). (C) Expression of p38 isoforms in primary osteoblasts (p38α/MAPK14; p38β/MAPK11; p38γ/MAPK12; p38δ/MAPK13) (n = 6 independent animals). (D) Calvarial protein levels of p38, phospho-p38, Creb, and phospho-Creb show impaired MAPK signalling in knockout animals (n = 3 independent animals) (*p<0.05; **p<0.01; ***p<0.001).

Efficiency of Cre activity, assessed by PCR analysis of calvarial DNA, stood at 50% to 80% (Fig. 1A). Osx1::Cre-mediated floxed recombination occurred exclusively in tissues that contain osteoblasts, whereas other tissues of mesenchymal origin retained intact floxed alleles (data not shown). In line with results from a previous report, body weight in male Osx1GFP::Cre mice was lower than in control littermates [20]. However, body weight of knockout mice was significantly lower than that of either control (FF) or Osx1-GFP::Cre mice (Figure S1). We further analyzed p38α expression in calvaria and tibia, as well as osteoblasts isolated from knockout mice and control littermates. In bone tissues and cultured osteoblasts we obtained a 60% reduction in p38α mRNA expression with no significant changes in the expression levels of the other p38 MAPK isoforms (Fig. 1B–C). Reduction in the p38α mRNA levels also resulted in decreased p38α protein expression in the calvaria of p38α-deficient mice (Fig. 1D). Interestingly, when phosphorylated levels of p38 isoforms or CREB (a target of p38 MAPK signalling) were analyzed, they had been reduced by about the same extent as p38α protein levels (Fig. 1D). These data suggest that the p38α isoform contributes to the total p38 MAPK signalling in mature osteoblasts.

### Cranio-facial and skeletal alterations in p38α knockout mice

**Figure 2:**
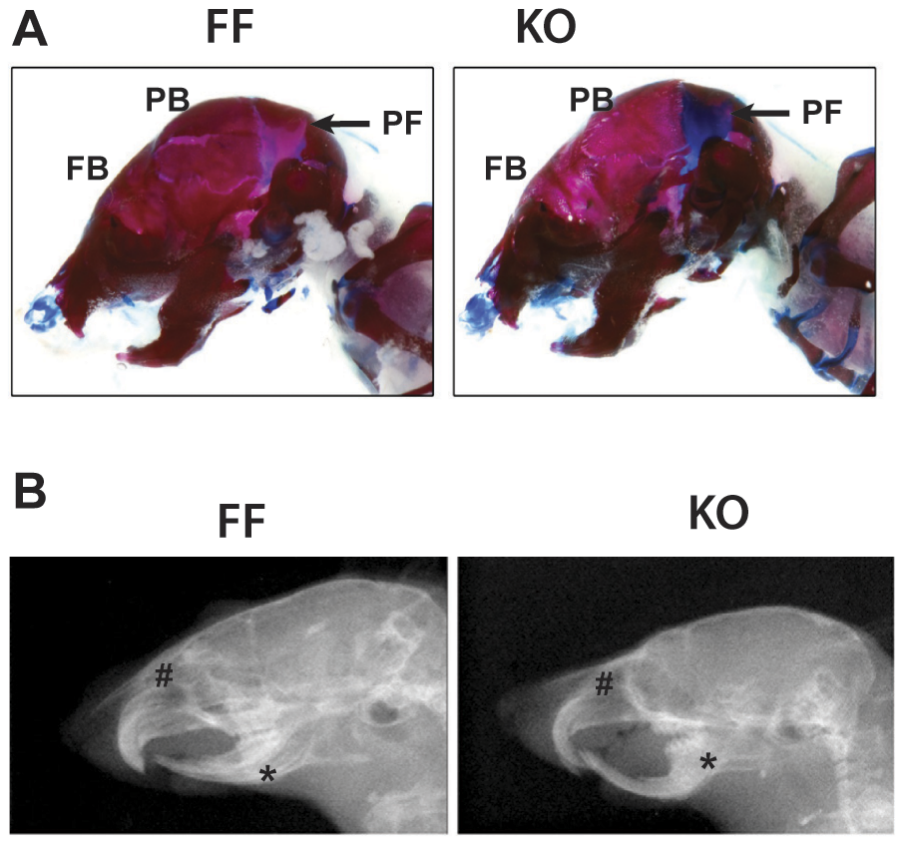
p38α deletion is associated with bone developmental defects. (A) Alcian blue/alizarin red staining of P7 mice pups. Alcian blue and alizarin red stain cartilage and calcified structures respectively (FB: frontal bone; PB: parietal bone; PF: posterior fontanel) (B) Radiographic lateral projections of 8-week old mice show maxilla and mandible hypoplasia (# marks maxilla and * marks mandible).

Skeletal preparations of newborn knockout mice showed hypoplastic cranio-facial bones compared to littermate controls In particular, impaired mineralization of frontal and parietal bones and delayed posterior fontanel ossification (Fig. 2A). Later, X-ray images further confirmed a hypomineralization of the calvaria and abnormal development of the maxilla and mandible in 8 week-old mice (Fig. 2B). Skeletal preparations showed no other obvious changes in the overall skeletal structure, although a small but significant decrease in the length of long bones was observed in knockout mice (femur length of knockout mice was 92% that of control mice). Both male and female knockout mice similarly displayed these cranio-facial phenotypes (data not shown).

**Figure 3:**
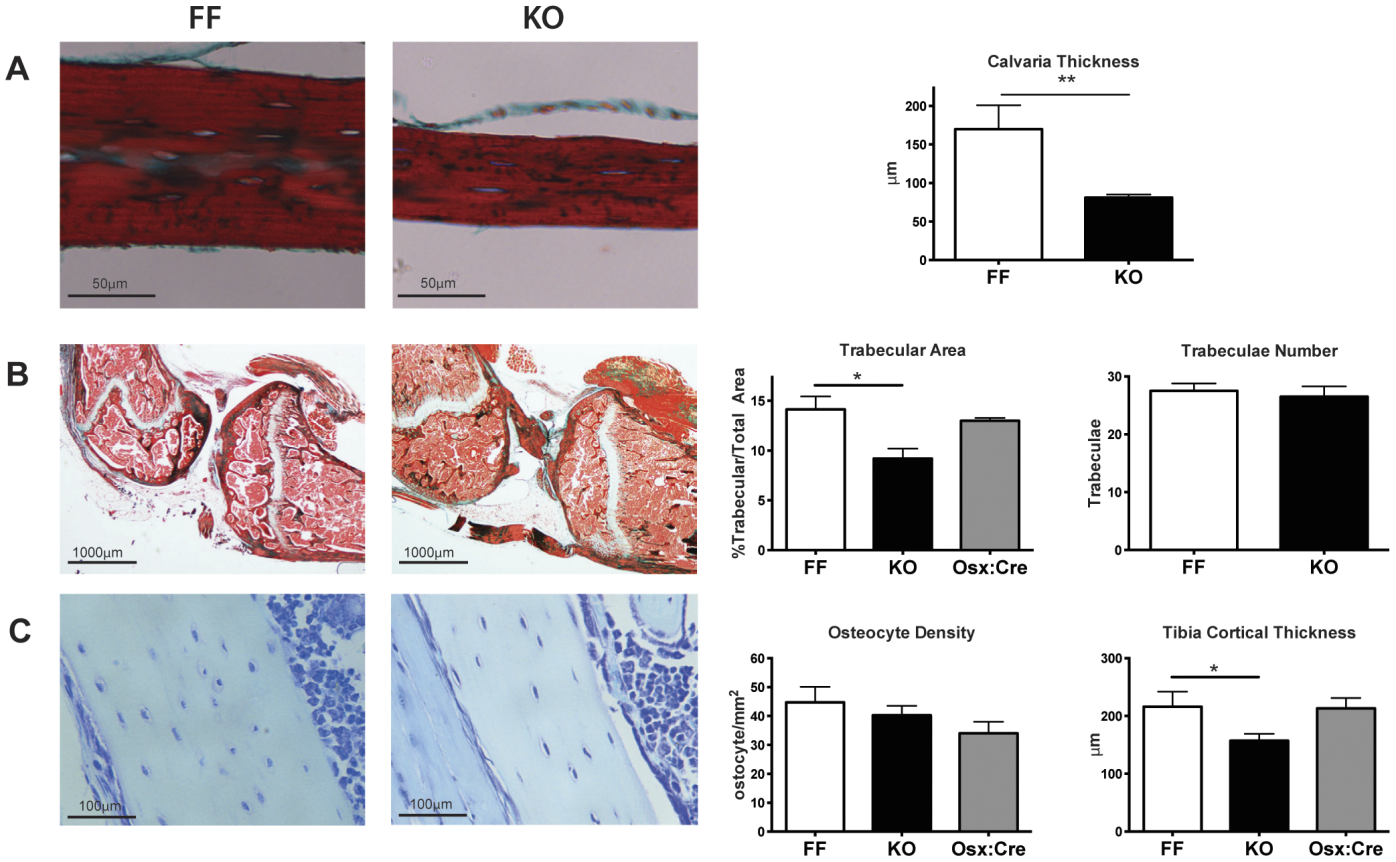
Histological analysis of bones from control and p38α knockout mice. (A) Representative images of calvarial sections stained with H&E. Right panel shows quantification of calvarial thickness (n = 5 independent animals). (B) Representative images of long bone sections stained with H&E. Quantification of trabecular area and trabecular number from tibiae sections are shown in the right panel (n = 3 (KO) and 4 (FF) independent animals). (C) Thickness and osteocyte count from cortical areas of long bone stained with toluidine blue (n = 3–6) (*p<0.05; **p<0.01; ***p<0.001).

Histological evaluation of calvaria from p38α knockout and control mice at 12 weeks of age showed a substantially lower thickness in knockout than in control mice (Fig. 3A). Similarly, staining of proximal tibiae and distal femurs showed thinner cortical bone and a significantly smaller trabecular area in tissues from knockout than in tissues from control or Osx1-GFP::Cre mice (Fig. 3B&C). These data indicate that deletion of p38α in osteochondroprogenitors leads to a strong decrease in bone mass of both intramembranous and endochondral origins. They also corroborate previous reports showing that the bone phenotype in young Osx1-GFP::Cre mice was overcome by 12 weeks of age, with no differences observed between Osx1-GFP::Cre and control mice [20], [21]. There was no change in the number of osteocytes per area but, since the cortical area in p38α-deficient tibiae was lower, the total number of osteocytes was also lower in cortical samples from knockout mice (Fig. 3C).

**Figure 4:**
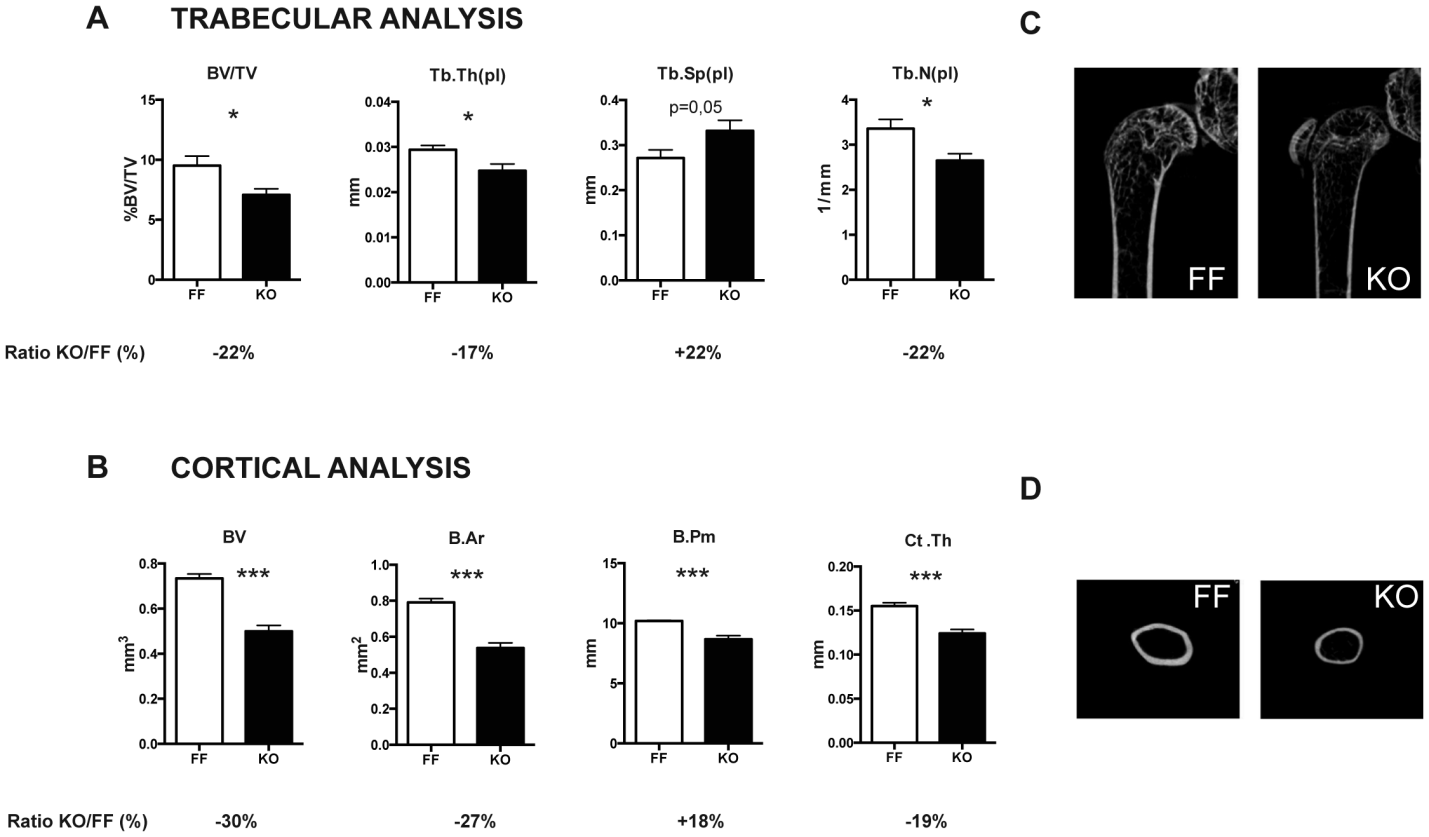
μCT evidences osteopenic bones in p38α deficient bones of 12-week-old mice. (A) The distal epiphysis of femurs from control and knockout littermates were analysed by μCT. Trabecular bone volume (BV/TV), trabecular number (Tb.N), trabecular thickness (Tb.Th) and trabecular separation (Tb.Sp.) were determined (n = 8 (KO) and 12(FF)). (B) Femur cortical bone analysis of 12-week-old male mice revealed that knockout mice had decreased cortical volume and thickness around the midshaft. Bone volume (BV), bone area (B.Ar), bone perimeter (B.Pm) and cortical thickness (Ct.Th) were determined (n = 6 (KO) and 12 (FF)) (*p<0.05; **p<0.01; ***p<0.001). Differences between values from KO and FF mice (expressed as %) are also included (C) Representative images of distal femur diaphysis and (D) femur cortical bone from control and knockout mice are also shown.

For more accurate measurements of bone structure, trabecular and cortical bone architecture were assessed by micro-computed tomography (μCT) in male mice. Collectively, the μCT quantitative results confirmed histological images. p38α-deficient adult mice at 12 weeks of age showed less trabecular bone volume in distal femurs associated with low trabecular thickness (17% less) and a significantly lower trabecular number (Fig. 4A). In addition, knockout mice also displayed decreased femoral cortical bone volume and thickness (30% and 19% reduction, respectively) (Fig. 4B).

### Disruption of p38α impairs osteoblast differentiation and function

**Figure 5:**
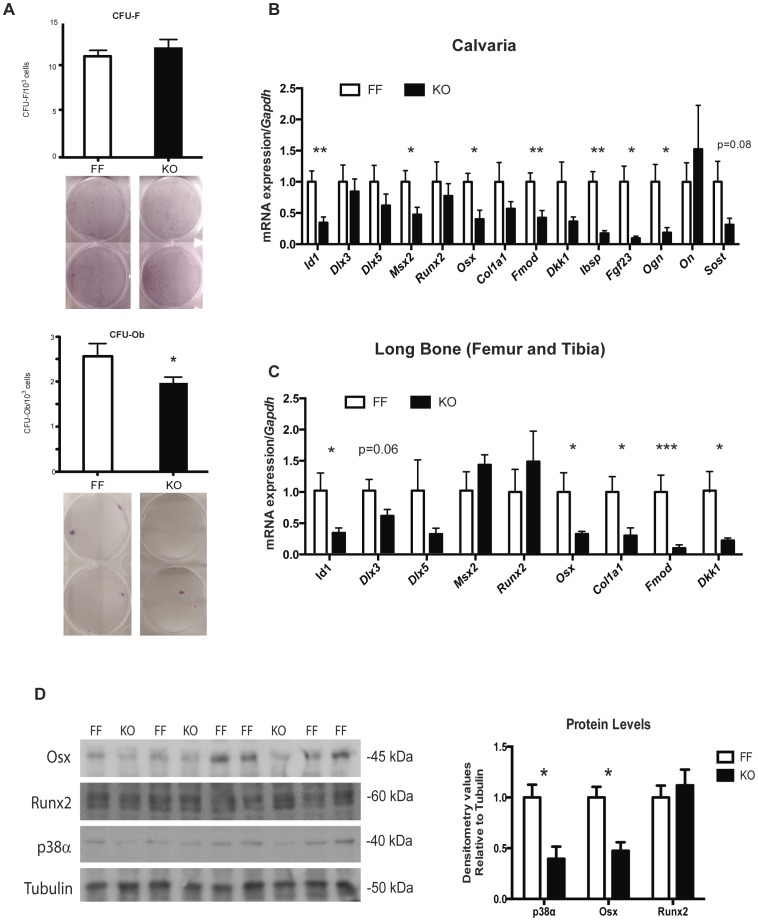
p38α deletion in osteoblasts affects the expression of osteogenic markers. (A) Graphs show fibroblast-colony forming units (CFU-F) (upper panel) and osteoblast-colony forming units (Ob-CFU) of mesenchymal stem cells from bone marrow of FF and KO long bones (n = 9 per genotype). Representative images are also shown. (B&C) qRT-PCR of mRNA extracted from calvariae (B) and long bones (bone-marrow flushed out tibiae and femurs) (C) of 12-week-old mice (n = 4 (KO) and 8 (FF) independent animals). (D) Protein levels of OSX and p38α were decreased in calvaria from knockout mice. Left panel: western blot of calvarial bones showing Osterix (OSX), RUNX2, p38α and alpha-TUBULIN as control. Right panel: graph depicting densitometric analysis of western blots (n = 3(KO) and 6(FF)) (*p<0.05; **p<0.01; ***p<0.001).

In order to discern if p38α affects osteoblastogenesis, we conducted colony-forming assays from bone marrow cultures. Undifferentiated mesenchymal cells from long bones did not show differences in fibroblastic colony forming units. After culturing them for 18 days in osteoblast differentiation medium, cell cultures were stained for alkaline phosphatase activity, showing a significant decrease (24% reduction) in KO alkaline phosphatase-positive colonies when compared to FF cultures (Fig. 5A). To further characterize the osteoblastic role of p38α, RNA was isolated from both the calvaria and bone marrow-flushed tibia of mice at 12 weeks of age. Expression of osteoblast genes from p38α-deficient mice and littermate controls was measured by qPCR (Fig. 5B,C). Expression of the early osteoblast differentiation marker *Col1a1* was decreased in calvaria and tibia (reaching a significantly lower value only in tibiae). In addition, expression of the late osteoblast differentiation markers *bone sialoprotein* (*Ibsp*), *fibromodulin* (*Fmod*) and *osteoglycin* (*Ogn*) [3], [22] fell to a greater extent, as did the known osteocyte markers *Dkk1*, *Fgf23* and *sclerostin* (*Sost*) [23] (Fig. 5B,C). These results indicate that progression of osteoblast differentiation *in vivo* is defective in knockout mice, beginning at an early stage and more dramatic at later stages.

To grasp the mechanisms for such transcriptional effects on osteogenic markers, expression of osteoblast-specific transcription factors was also analyzed at 12 weeks of age. Expression levels of *Dlx3*, *Dlx5* or *Runx2* were, at most, only slightly lower in tibiae and calvarial tissues of knockout mice and *Msx2* was only significantly lower in calvarial samples. However, *Id1* or *Osx*, which are induced later in development [13], [24], [25], showed significantly lower expression in both calvaria and tibiae of knockout mice (60% less than in control mice). Reduction in the *Osx* mRNA levels also resulted in lower OSX protein levels in the calvaria of p38α-deficient mice, whereas there was no change in RUNX2 protein levels (Fig. 5D).

**Figure 6:**
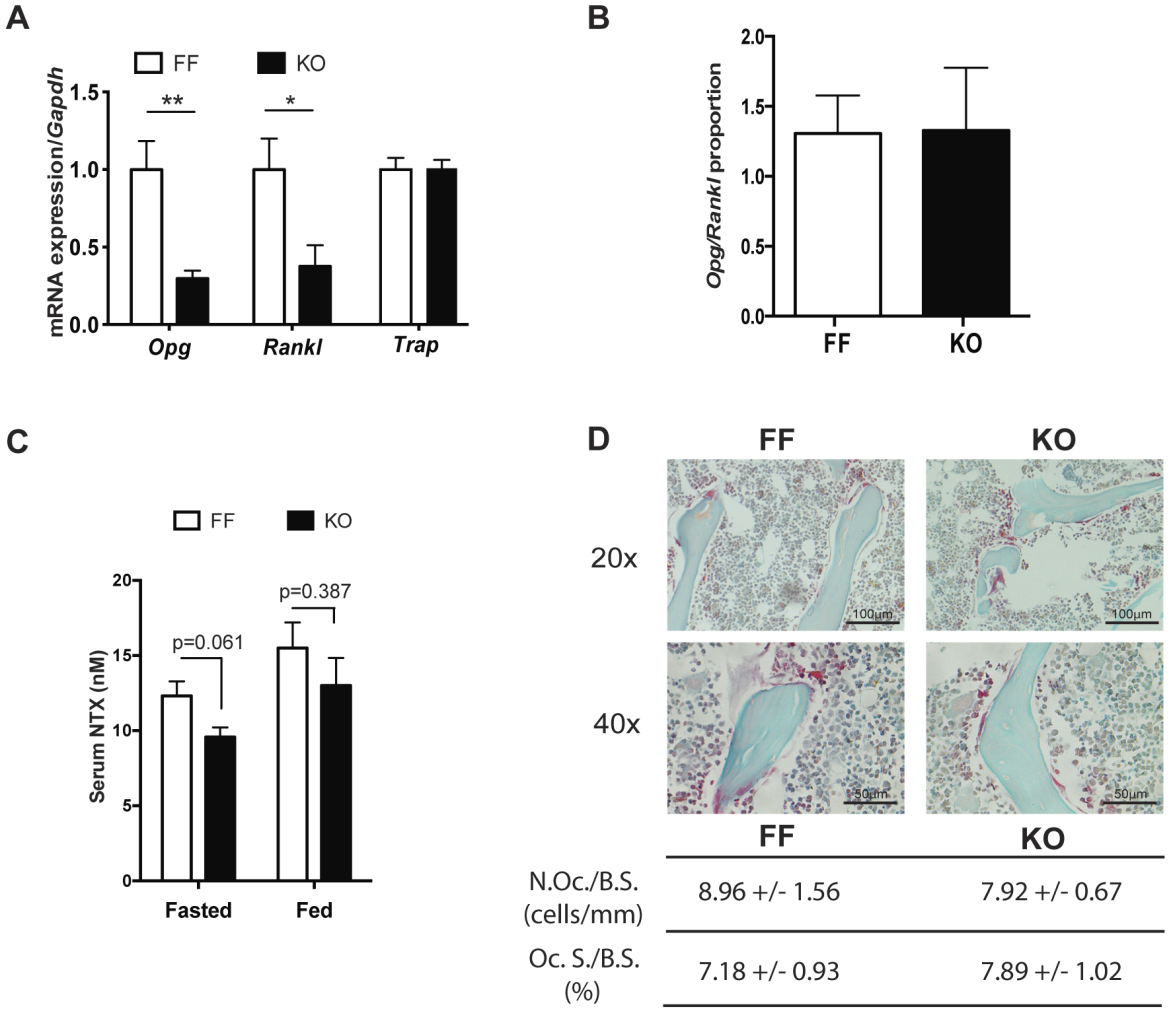
p38α blockage does not affect bone resorption parameters. (A) *Rankl*, *Opg* and *Trap* mRNA levels in long bones from knockout and control mice (n = 5 (KO) and 8 (FF) independent animals). (B) The *Rankl/Opg* expression ratio was not modified. (C) N-terminal telopeptides of collagen from serum of 12-weeks-old mice do not display significant differences between control and knockout mice (n = 4 (KO) and 10 (FF) independent animals). (*p<0.05; **p<0.01). (D) TRAP staining of proximal tibiae from FF and KO mice (n = 3 per genotype) showed no differences in osteoclast parameters (N.Oc./B.S., numbers of osteoclast/Bone perimeter; Oc. S./B.S, osteoclast surface/Bone perimeter). Data is presented as means ± SEM. Image shows representative images counterstained with fast green (20× and 40× magnification).

p38α-deficient mice did not show significant changes in the serum alkaline phosphatase levels, a known marker of bone formation [26]. Similarly, calcium and phosphate levels in serum and urine remained unchanged between knockout and control littermates (Figure S2). *Rankl* and *Opg* mRNA expression was analyzed in tibiae from mice at 12 weeks of age to determine whether increased osteoclastogenesis could also explain the lower bone mass of knockout mice. Both *Rankl* and *Opg* expression were lower in knockout mice. However, the relative *Rankl/Opg* ratio, which determines osteoclast activation [27], was not significantly altered (Fig. 6A,B).Similarly, expression of the osteoclast marker *Trap* (tartrate-resistant acid phosphatase) was not modified (Fig. 6A). To further explore changes in bone resorption, we measured cross-linked N-terminal telopeptides of type I collagen (NTX) levels in serum of fed and fasted mice [28]. NTX levels were slightly lower in serum of p38α-deficient mice, although they did not reach significant variation (p = 0.061) (Fig. 6C). Moreover, specific staining for TRAP activity in tibiae preparations did not show gross differences in the number of positive cells or in the ratio between osteoclast surface and trabecular bone surface (Fig. 6D). These results indicated that p38α inactivation in osteoblasts did not increase osteoclast function, and that the reduced bone mass in these mice was not due to increased bone resorption.

**Figure 7:**
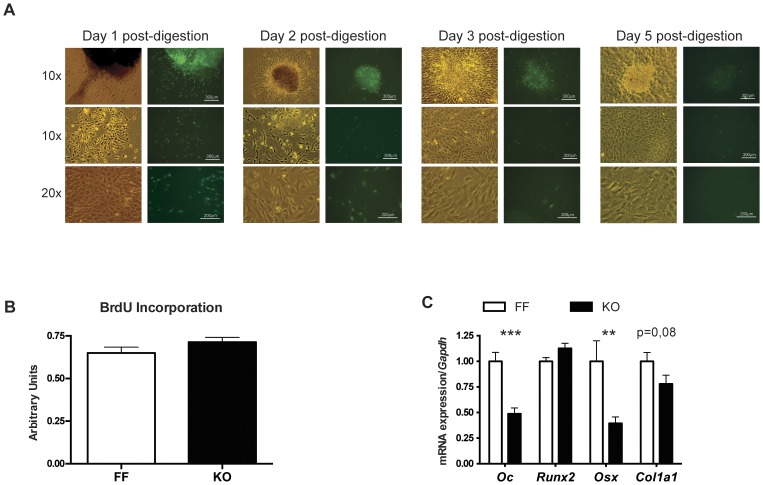
*In vitro* analysis of primary cultures reproduces the gene expression disturbances seen *in vivo*. (A) Images show bone chips and isolated osteoblasts from KO animals after collagenase treatment. From left to right: 1, 2, 3 and 5 days post-digestion. Endogenous GFP expression from the Osx1-GFP::Cre transgene is lost as culture advances. (B) BrdU incorporation on primary osteoblasts cultures. (C) qRT-PCR reveals the comparative mRNA expression levels of *Col1a1*, *Bglap* (OC), *Osx* and *Runx2* in primary cultures of osteoblasts from control and knockout pups (n = 6 independent animals per genotype). (*p<0.05; **p<0.01).

To further explore the cell mechanism involved in the bone phenotype of p38α-deficient mice, we isolated and analyzed primary osteoblasts *in vitro* from knockout and control littermates. In isolated calvaria, as well as freshly isolated osteoblasts from knockout mice, expression of the EGFP-Cre transgene was evident in almost all osteoblasts (Fig. 7A). Once the osteoblasts were cultured in vitro, EGFP-Cre expression declined over time. Osteoblasts from knockout or control mice showed no significant differences in their proliferation rate under media with or without fetal bovine serum as mitogenic stimulus (Fig. 7B). Osteoblasts from knockout and littermate controls were also cultured to confluence in osteogenic media for 10 days and their RNA was extracted and analyzed. As previously found for calvaria and tibia samples, *Runx2* expression was unaltered and *Col1a1* expression was slightly lower without reaching significance, whereas the markers of late differentiation *osteocalcin* (*Bglap*) and *Osx* showed significantly lower values than osteoblast cultures from control animals (Fig. 7C). Taken together, these results indicate that p38α-deficient osteoblasts have a cell-autonomous defect in differentiation potential and further osteogenic function.

### Skeletal alterations after deletion of p38α in adult mice

**Figure 8:**
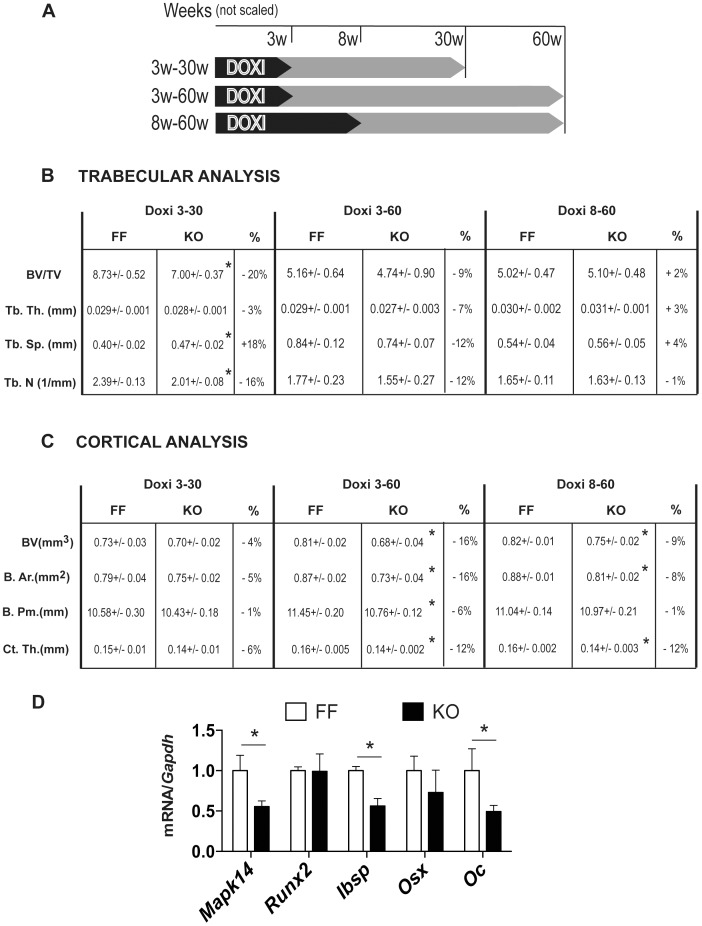
p38α is necessary for adult bone homeostasis. (A) Scheme of doxycyline (doxi) administration and sacrifice of the different mice groups analyzed. (B) The distal femoral epiphysis of control and knockout mice after different time of doxycycline (doxi) treatments (3 or 8 weeks) were analyzed by μCT at 30 or 60 weeks of age. Trabecular bone volume (BV/TV), trabecular number (Tb.N), trabecular thickness (Tb.Th) and trabecular separation (Tb.Sp.) were determined (n = 5 (KO) and 8 (FF)). (C) Cortical bone volume (BV), bone area (B.Ar), bone perimeter (B.Pm) and thickness (Ct.Th) analysis was performed in femur midshafts from these animals (n = 5–8). Data are presented as means ± SEM. (*p<0.05). Differences between values from KO and FF mice (expressed as %) are also included. (D) qRT-PCR showing gene expression of calvariae from 30-week old FF and KO mice treated with doxycycline until 3 weeks of age (Doxi 3–30). Graph shows levels of *p38a* (*Mapk14*), *Runx2*, *bone sialoproteïna* (*Ibsp*), *osterix* (*Osx*) and *osteocalcin* (*Oc*) (FF n = 4, KO n = 5) (*p<0.05; **p<0.01).

To evaluate the role of p38α in fully formed bones, doxycycline was delivered in drinking water to pregnant mothers and to newborn mice until 3 or 8 weeks of age. Doxycycline represses the Osx1-GFP::Cre promoter and recombination at the p38α locus (exons 2–3) is prevented until doxycycline is removed from drinking water [19]. Mice that received doxycycline until 3 weeks after birth were killed at 30 or 60 weeks of age and those that received doxycycline until 8 weeks of age were killed at 60 weeks (Fig. 8A). qRT-PCR analysis showed that p38α expression was reduced (60% compared to control) in bones of mice at 30 or 60 weeks of age (Fig. 8D). μCT analysis was performed on distal femurs of these mice. Whereas we found no major changes in cortical parameters with age in control animals, there was an age-dependent 42% decrease in trabecular bone volume accompanied by a decrease in trabecular number in control animals by 31% (Fig. 8B,C and Figure S3). These results fully corroborate previous data on C57BL/6 mice [29]. Cortical analysis at the femoral mid-shaft showed that deletion of p38α three weeks postnatally results in minor differences in cortical bone parameters after 30 weeks. However, 60 weeks after p38α deletion bone volume was lower (16% reduction) due to a significant decrease in cortical thickness (Fig. 8C and Figure S3). Similarly, deletion at 8 weeks after birth and analysis at 60 weeks showed lower bone volume (9% less in KO animals) and a reduction in cortical thickness (12% thinner). These results suggest that postnatal deletion of p38α results in cumulative effects over time on cortical bone turnover.

In contrast to the late effects (60 weeks) of p38α deletion on femoral cortical thickness, the effects in trabecular bone were more clearly seen at early ages. Deletion of p38α three weeks postnatally led, after 30 weeks, to reduced bone volume and trabecular number (20% and 16% reduction, respectively) but no changes in trabecular thickness (Fig. 8B). However, after 60 weeks there were no significant changes in the trabecular bone parameters in animals with p38α deletion at either 3 or 8 weeks (Fig. 8B and Figure S3). These data suggest that the effects of age-dependent reduction on trabecular bone could make it difficult to detect differences between groups [30]. Alternatively, progressive age-dependent reduction in trabecular number could dampen the p38α deletion effects in order to maintain some biomechanical competence. Moreover, our data reinforce the idea that bone remodelling has different patterns on cortical and trabecular surfaces [29], [31], [32]. We also analyzed the expression of osteoblast markers in animals at 30 weeks of age and treated them with doxycyline until 3 weeks of age. qRT-PCR data showed invariant levels of *Runx2* mRNA but a significant decrease in the late osteogenic markers *Ibsp*, *Osteocalcin* and *Osx* (Fig. 8D)

## Discussion

Recent studies have documented the importance of p38 signalling in skeletal development *in vivo*. Mice in which TAK1, MKK3 or MKK6 were deleted in osteochondroprogenitors from early development had low bone mass phenotype due to impaired osteoblast differentiation [7]. Similarly, when p38α deletion in osteoblasts occurred during late embryogenesis, mice developed a progressive decrease in bone mineral density (BMD) [18]. These effects also appeared with a different temporal pattern. For instance, inactivation of p38 signalling in osteochondroprogenitors (Osx1-GFP::Cre) (deletion around E14–15 [19]) resulted in skeletal defects in newborn mice, whereas inactivation in late embryogenesis (E-16.5–18.5) in Ocn-Cre showed no significant alterations before five weeks of age [7], [18], [33].

Our results confirm that p38α is critical for early bone formation and development; they also demonstrate this requirement for bone homeostasis in adulthood. We took advantage of an inducible Cre system that controls the onset of p38α disruption after birth by removing doxycycline from drinking water [19]. We performed the deletion of p38α at 3 weeks of age, when the bone architecture is already established, and BMD and cortical thickness is about 60–70% of that of adult mice, or at 8 weeks of age, when mineralization in cortical and trabecular bone has already reached peak values [29], [31], [34], [35]. Significant trabecular bone loss was detected at 30 weeks of age. However, at 60 weeks of age cortical bone volume and thickness were lower while differences from control mice in trabecular bone volume and number no longer persisted in p38α knockout animals. In confirmation of our results, most previous studies in aged mice in B6 background established that trabecular bone loss was mostly dependent on complete elimination of individual trabeculae, whereas trabecular thickness was not affected and sometimes even increased [29], [36], [37]. This effect mirrors morphological traits of osteoporosis in humans [38], [39]. In our model, p38α had a significant effect on bone remodelling after 30 weeks of age. One can speculate that, later on, these effects could be tempered by the age-dependent reduction in trabecular bone, to maintain some of the latter's biomechanical competence in face of decreasing cortical strength. Similar mechanisms of compensation in aged mice have been suggested in different models of bone loss in rodents [36], [37], [40]. When p38α was deleted after peak bone values, the cumulative reduction in cortical bone volume and thickness became apparent only after 60 weeks of age. Thus, in addition to its role in early skeletogenesis and mineralization, p38α is also essential for differentiation of osteoblasts and/or for osteoblasts to maintain their function in mineralized adult bone. As a result of this, our data also show that efficient bone remodelling throughout life requires p38 function and that inhibition of this signalling pathway would result in significant osteoblast malfunction during chronic treatments. Several chemical p38 MAPK inhibitors have been developed and envisaged as potential autoimmune or anti-inflammatory drugs as well as inhibitors of neuropathic pain [41], [42]. Our data support the view that osteoporotic side-effects should be taken into account, even for highly selective p38 kinase inhibitors. Similarly, the effects of p38 signalling on the bone resorption activity of osteoclasts should also be analyzed. Inhibition of p38 signalling in osteoclasts has been shown to reduce their resorptive activity and improve trabecular bone loss induced by estrogen deficiency [10].

Bone remodelling depends on a highly coupled balance between osteoblast and osteoclast functions. Even though osteoblast function was defective, no significant differences in *Rankl/Opg* ratio or in TRAP staining of tibiae were found and only a slight, but not significant, decrease in serum NTX was obtained in p38α-deficient mice. These results indicate that impaired osteoblast function due to the lack of p38α did not severely modify osteoclast function. Moreover, the effects of p38α deletion were also marked during differentiation of osteoblast cultures *in vitro* or osteoblastogenesis from mesenchymal stem cells, confirming that changes in osteoblast function are cell-autonomous and do not rely on inaccurate osteoblast-osteoclast communication.

p38α activation is normally associated with anti-proliferative functions, since negative regulation of proliferation has emerged as a highly conserved function of p38α in various types of primary cells [43]. We could not find changes in osteoblast proliferation and/or apoptosis that could explain their lower function *in vivo*. Similar absence of effects on proliferation and/or apoptosis has been demonstrated in vitro in mesenchymal osteoblast precursors [9], [44]. Thus, our data imply that p38α-deficient osteoblasts have a cell-autonomous defect in differentiation potential that impairs their osteogenic and mineralizing function. Moreover, additional effects of p38α deficiency in bone turnover could involve the ability of p38 signalling to modulate the motility of osteoprecursors and osteoblasts. Although not addressed in this report, migration of mesenchymal stem cells *in vitro* has been shown to depend on p38 kinase activity and mouse models of impaired motility of osteoblasts also result in reduced bone mass [45], [46], [47]

It was previously suggested that, since only mice with deletion at MKK6, and not MKK3, had calvarial hypomineralization, signalling from distinct p38 isoforms could differently affect intramembranous and endochondral ossification [5]. Furthermore, although whole-body deletion of p38β in mice was reported to have no major phenotype, it was later shown that they had osteopenia of long bones [7], [48]. Our results indicate that p38α is an indispensable isoform involved in both intramembranous and endochondral bone development. Deletion of p38α during embryogenesis affected intramembranous calvarial bone development as early as on postnatal day 7. Similarly long bones arising from endochondral ossification also showed an important reduction in both trabecular and cortical bone at very early developmental stages. So far no major differences in substrates and transcriptional targets activated by p38α or p38β have been shown, suggesting their functional redundancy [49]. It could thus be hypothesized that the relative role of these two subunits would largely depend on their differential tissue expression and/or activity in osteoblast cells.

Mechanistically, in our mice model, lack of p38α leads to similar changes in the expression of osteoblast-specific transcription factors in calvaria, long bone and *in vitro* osteoblast cultures. Whereas expression levels of *Dlx3*, *Dlx5* or *Runx2* did not show significant changes and *Msx2* decreased only in calvaria, *Osx* levels decreased in all three conditions. These results corroborate *in vitro* data for which chemical inhibition of p38 signalling did not change *Dlx3*, *Dlx5* or *Runx2* expression, but blocked the transcriptional induction of *Osx* expression by BMP2 [13]. OSX have been shown to regulate transcriptionally the expression of *Col1a1*, *Ibsp*, *Bglap* and *Dkk1* [14], [15], [50], [51], [52]. Lower levels of OSX could account for impaired osteoblast maturation and function, through decreased transcription of these osteoblast and/or osteocyte genes. Furthermore, evidence demonstrates that several osteoblast-determining transcription factors are substrates of p38 and ERK MAPKs. It had previously been shown that DLX5, RUNX2 and OSX are substrates of p38 and multiple phosphorylated serines were identified [7], [13], [14], [16]. Phosphorylation of these factors promotes increased transcriptional activities for all of them through better recruitment of the transcriptional cofactor p300/CBP or the SWI/SNF subunit Brg1 [7], [13], [15]. Therefore, phosphorylation of DLX5, RUNX2 and OSX by p38 signalling constitutes an integration point, at which extracellular stimuli converge in the regulation of this MAPK. As a result, p38-dependent phosphorylation promotes their transcriptional activity and induces osteoblast maturation and function in bone remodeling and homeostasis. Our results agree with previously demonstrated requirements of OSX function for skeletal maintenance and osteoblast and osteocyte function during adulthood [53].

In summary, our data demonstrate that p38α is necessary both in early skeletogenesis, as well as in the maintenance of osteoblast differentiation and function during bone remodeling in adult life. Loss of p38α in osteoblasts impairs the expression and phosphorylation of osteoblast-specific transcription factors, blocking further osteoblast maturation and function. Our data also emphasize that clinical development of p38 inhibitors should take into account their potential effects on bone.

## Materials and Methods

### Generation of conditional *Mapk14* knockout mice

To delete p38α (*Mapk14*) specifically in osteochondroprogenitors, mice carrying loxP sequences flanking p38a alleles (a generous gift from Drs. Nebreda and Dr Pasparakis [54]) were crossed with Osx1-GFP::Cre (Osx:Cre) [19]. As the resulting *Osx1-GFP::Cre*;*Mapk14^f/f^* (KO) were fertile and born in the anticipated Mendelian ratio, they were crossed with *Mapk14^f/f^* (FF) to generate sibling control and KO mice. The Osx-Cre mouse line contains a tTA and a tetracycline-responsive element that allows the expression of Cre recombinase only in the absence of doxycycline. By maintaining the animals under a doxycycline regime (0.2 mg/ml in drinking water) we were able to control the timing of p38α excision. Three different regimes were examined: (1) doxycycline during pregnancy; (2) either until 3 weeks or until 8 weeks of postnatal life; (3) no doxycycline treatment to pregnant mothers or newborns (Fig. 8A). The mice were housed under controlled conditions (12 h-light/12 h-dark cycle, 21°C, 55% humidity) and fed *ad libitum* with water and a 14% protein diet (Teklad 2014, Harlan). Unless otherwise stated, all experiments were performed in male mice. All animal protocols were approved by the Ethics Committee for Animal Experimentation of the University of Barcelona (Barcelona, Spain).

### Genotyping (PCR)

Total DNA was extracted from a 3 mm piece of mouse tail. The Osx1-GFP::Cre transgene was identified by PCR using the following primers: OsxCre1: 5′-CTC TTC ATG AGG AGG ACC CT and OsxTGCK: 5′-GCC AGG CAG GTG CCT GGA CAT giving a resulting PCR band of 510 bp. The Mapk14 lox-P cassette was identified by the primers FloxX: 5′-CTACAGAATGCACCTCGGATG and FloxY: 5′- AGAAGGCTGGATTTGCACAAG (resulting bands of 188 for the floxed allele and 121 for the wild-type). Effective recombination was assessed by PCR of bone samples using the Flox X primer and the FloxZ: 5′-CCAGCACTTGGAAGGCTATTC, resulting in a band of 411 bp.

### Immunoblot analysis

Protein lysates were prepared from bone samples homogenized with a Polytron device in 50 mM Tris, pH 6.8, 10% glycerol and 1% SDS, separated by SDS-polyacrylamide gel electrophoresis, transferred to nitrocellulose and analyzed by immunoblotting. Membranes were incubated with specific antibodies for p38α (1∶1000 Cell Signaling), p-p38 (Cell 1∶1000 Cell Signaling), p-CREB (1∶1000 Cell Signalling), OSTERIX (1∶1000 Abcam) RUNX2 (1∶1000 MBL) and α-TUBULIN (1∶5000 Sigma). Horseradish peroxidase-conjugated anti-rabbit and anti-mouse secondary antibodies were used (GE).

### Primary osteoblast cultures and CFUs

Primary osteoblasts were seeded in culture after collagenase digestion of calvariae from P1–P4 mice pups. Bones were dissected from euthanized pups and their sutures and soft tissue were discarded. 8–12 calvariae were pooled per genotype and serially digested in a trypsin (0.025%)/collagenase II solution (1 mg/ml). The product of the first 5 minutes of digestion was discarded, while the product of a double 40-minute digestion was centrifuged (1200 rpm, 5 min.) and seeded on 60 mm culture plates. Primary osteoblasts were cultured in α-MEM with10% Fetal Bovine Serum (FBS) and penicillin-streptomycin (P-S). The cultures were expanded and used between passages 2–3. For colony-forming units (CFUs) assay bone marrow cells were flushed from tibiae and femurs from 8-week old FF and KO mice. Cells were seeded in DMEM with 10%FBS and the medium was replaced every 3 days. When the cultures reached 70% of confluence, attached cells were tripsinized for 3 minutes at room temperature and expanded. Then 5000 cells were seeded per well (6-well plate). For CFU-Fibroblasts, cultures were fixed and stained with crystal violet solution (0.2% crystal violet, 2% ethanol) for 30 minutes at 37°C. Then wells were washed with tap water and air-dried. Colonies (greater than 50 cells) were counted. For CFU-Osteoblasts, cells were grown in osteoblast differentiation medium (α-MEM, 10%FBS, 10 mM beta-glycerophophate, 50 µg/ml vitamin C, 1 nM dexamethasone and P-S) for 18 days. Colonies were stained for alkaline phosphatase activity using alkaline phosphatase kit from Sigma (86-R). Positive ALP colonies (bigger than 50 cells) were counted.

### Radiographic and μCT analysis

For conventional radiography 8 week-old mice were anesthetized with isofluorane and lateral radiographies were taken with a portable device. For μCT mice were euthanized at different ages and their hind limbs were dissected and cleaned of soft tissue. Femur and tibia were fixed in 4% PFA for 24 hours and stored in PBS with sodium azide at 4°C until the analysis. The μCT image was acquired through an aluminum filter of 1 mm, with the samples in air in a SkyScan 1076 High resolution in-vivo micro-CT scanner (SkyScan, Kontich, Belgium). Selection of the scan energy and voxel size was based on optimizing the requirements of scanning time and tissue detail. The following conditions were used: 9 µm isotropic voxel size was used, at 50 kV, 200 µA with an exposure time of 1600 ms and 180° rotation. Scans were reconstructed using the *Recon* software provided by *SkyScan*. For trabecular measurements, a 1 mm-diameter circular VOI was employed, starting at 100 slices from the distal growth plate of the femur and extending to the diaphysis for 150 slices. Cortical measurements were computed manually delineating the femur medial cortex for 100 slices around the femur midshaft. A Gaussian noise filter was applied for the reconstruction. The *CTscan SkyScan* software was used for image analysis A global binary threshold was manually established at 25 for trabecular analysis and 155 for cortical analysis.

### Histological preparations

For histological preparations, samples were fixed in 4% paraformaldehyde for 24 h at 4°C, decalcified in EDTA and HCl for 2–4 days at 4°C and processed for paraffin embedding. Samples were cut in 4 µm sections and stained with toluidine blue, Masson's trichrome or hematoxylin/eosin. To reveal changes in cartilage and bone of the entire skeleton, P7 newborn mice were sequentially stained with alcian blue (0.015% alcian blue 8GX) and alizarin red (0.005% alizarin sodium sulfate) after fixation with 95% ethanol. Samples were cleared with 1% KOH and maintained in glycerol∶ethanol (1∶1). For TRAP staining, long bone samples were decalcified in EDTA for 10 days and processed for paraffin embedding. Deparaffinied slides were prewarmed with basic incubation solution (sodium acetate, sodium tartrate and glacial acetic acid) for 30 minutes at 37°C. Then, naphtol-ether substrate (naphtol AS-BI phosphate, 2-ethoxiethanol) was added. After 1 hour incubation at 37°C, slides were placed in a new bucket containing basic incubation buffer plus sodium nitrite and pararonsaniline dye (pararosaniline dye in 2 N HCl) in order to develop specific osteoclast staining (5 minutes, 37°C). Preparations were counterstained with 0.02% fast green for 45 seconds. 3 different sections from 3 independent animals were analyzed per genotype.

### qRT-PCR

Bone samples were dissected from euthanatized animals and immediately frozen in liquid nitrogen. Prior to congelation bone marrow was removed from long bones. For that purpose, epiphysis were cut and discarded and, by means of a 26G-needle, PBS was flushed through the diaphysis until bone marrow was removed. Bone samples were individually homogenized using a Polytron device and RNA was extracted from bone or cell samples by TriSure (Bioline). RNA from tissue samples was extracted and processed individually. A minimum of 4 samples (from different animals) per group was considered for each experiment (*n* stated in Figure Legends). At least 2 µg of purified RNA were retrotranscribed using the High Capacity Retrotranscription Kit (Applied Biosystems). Quantitative PCRs used an ABI Prism 7900 HT Fast Real-Time PCR System and a Taqman 5′-nuclease probe method or customized TLDA arrays (Applied Biosystems). 50 ng of cDNA were used per reaction (two replicates per sample) on a 384-well plate. All transcripts were normalized to *Gapdh* expression.

### BrdU incorporation and serum analysis

For the BrdU incorporation assay 2000 cells per well were seeded in a 96-well culture plate. 24 hours later, BrdU labeling solution was added for 4 hours. BrdU incorporation into DNA was quantified with the Cell Proliferation ELISA kit (Roche). Blood was collected from posterior vena cava. It was left to clot for 1–2 h at room temperature and centrifuge at 1000G to obtain blood serum, which was immediately frozen at −80°C for posterior analysis. Cross-linked N-telopeptides of type I collagen (NTX) in mouse serum were measured by ELISA kits (Cusabio Biotec, CO). Alkaline Phosphatase (ALP) levels were determined following the recommendations of the International Federation of Clinical Chemistry. For calcium and phosphate concentration, the arsenazo III and molibdate methods were applied respectively. ALP, calcium and phosphate levels were analyzed at the Clinical Biochemistry Service of the Faculty of Veterinary Medicine (Universitat Autònoma de Barcelona) using an Olympus AU400 analyzer.

### Statistical analysis

The Student's *t* test was employed for statistical analysis. Quantitative data were presented as means ± SEM. Differences were considered significant at p values of less than 0.05: *p<.05, **p<.01, and ***p<.001.

## Supporting Information

Figure S1 Weight progression curves show decreased weight gain in knockout mice compared to control or Osx1-GFP::Cre mice along 18 weeks (n = 9 (KO) and 18 (FF)).
(TIF)

Figure S2 Calcium and phosphate levels remain unchanged. (A) Table shows blood serum levels of calcium (Ca), phosphate (Pi) and alkaline phosphatase from 12 week-old FF and KO mice. Data show no differences between FF and KO, either in fed (p>0,05) or fasted state (p>0,05) (n = 13–16). (B) Urine levels of calcium and phosphate are shown relative to creatinine (Cr) excretion levels (n = 9–16). (*p<0,05; **p<0,01).
(TIF)

Figure S3 Representative images of distal femur diaphysis and femur cortical bone from control and knockout mice subjected to the distinct doxycycline administration and sacrifice regimes.
(TIF)
