# Supplementary figures and images for: The p38α MAPK Function in Osteoprecursors Is Required for Bone Formation and Bone Homeostasis in Adult Mice
Source: PLoS One. 2014 Jul 9;9(7):e102032. doi: 10.1371/journal.pone.0102032 (PMC4090229; doi:10.1371/journal.pone.0102032)

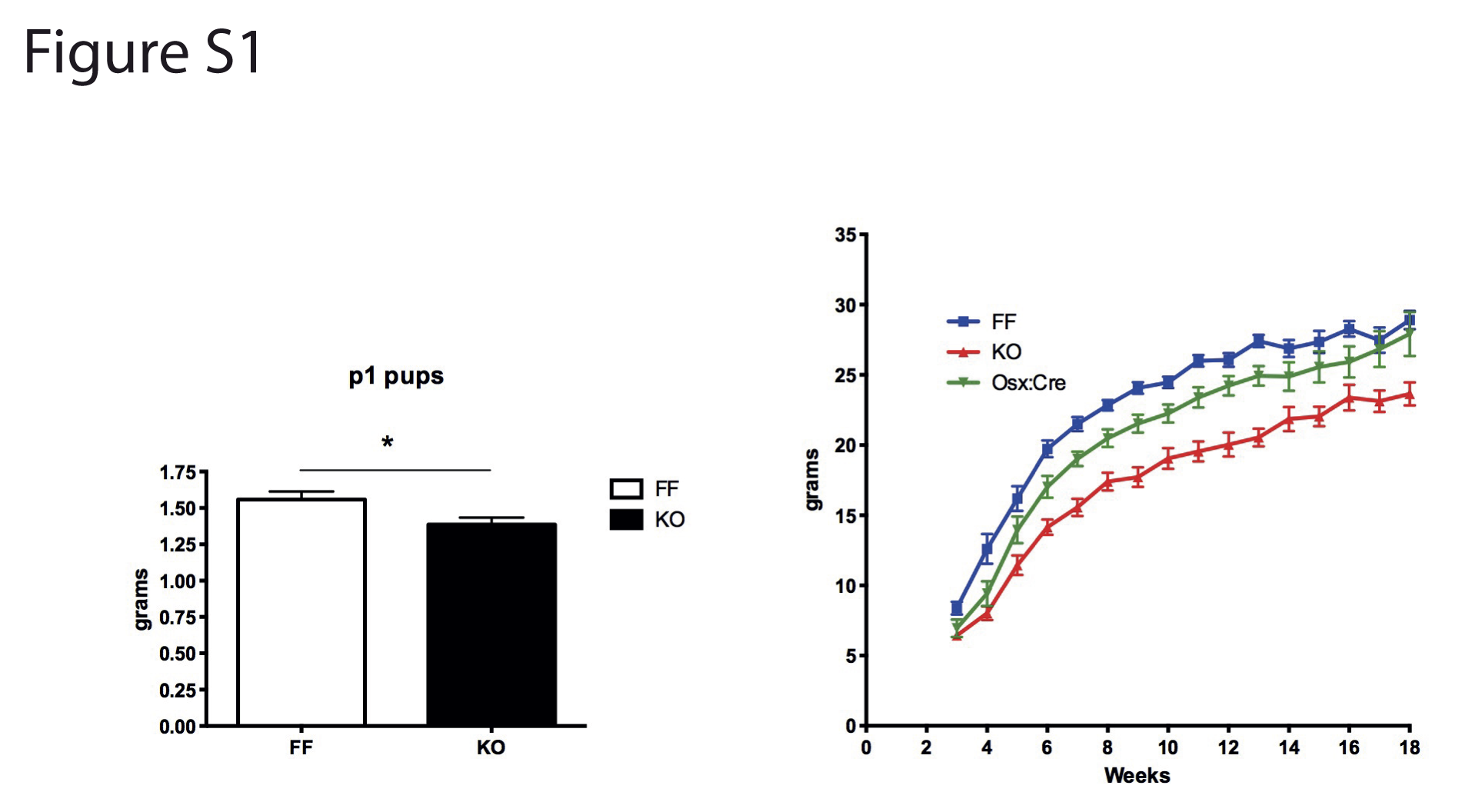

Supplement: Figure S1 — Weight progression curves show decreased weight gain in knockout mice compared to control or Osx1-GFP::Cre mice along 18 weeks (n = 9 (KO) and 18 (FF)). (TIF) [file pone.0102032.s001.tif]

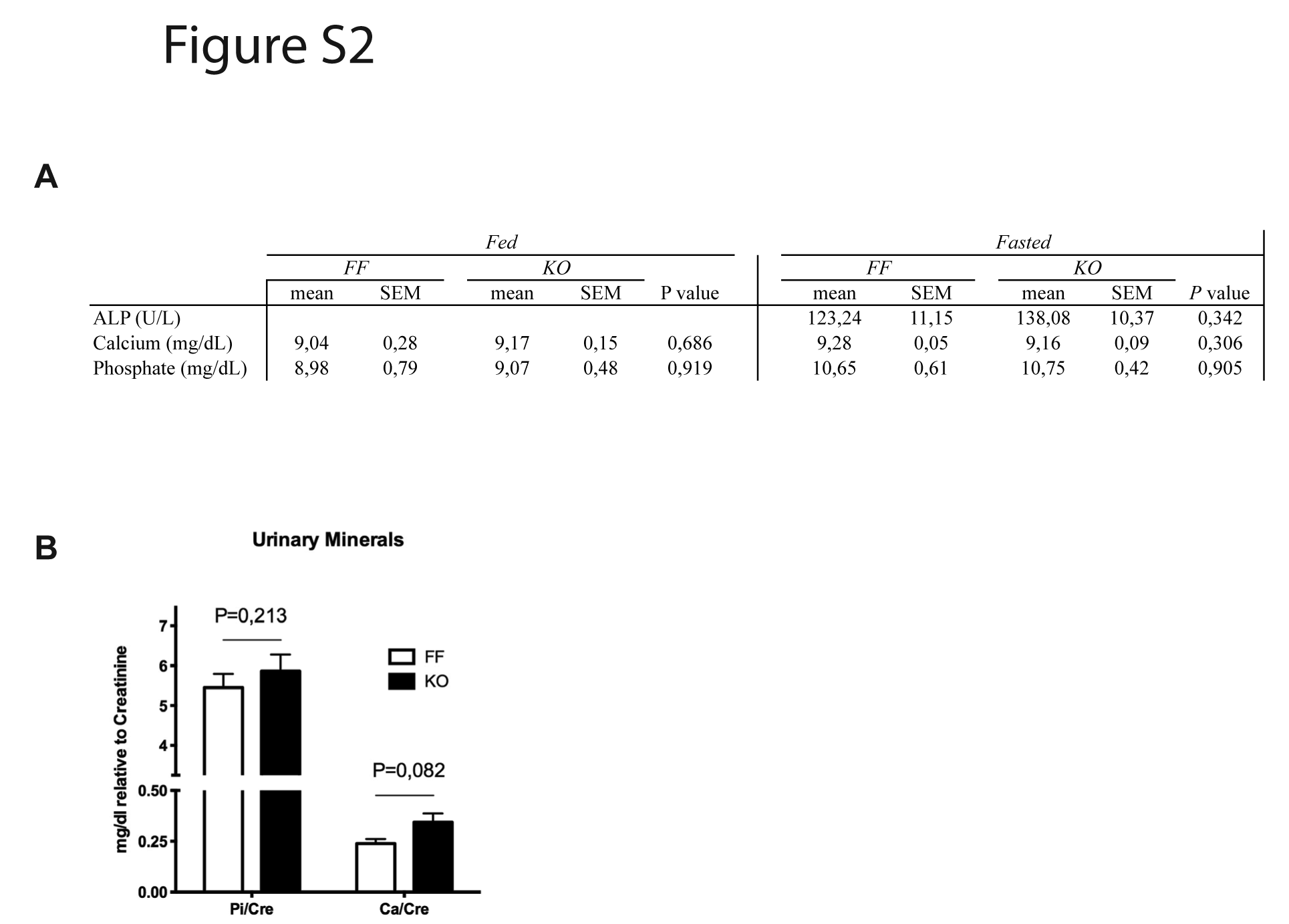

Supplement: Figure S2 — Calcium and phosphate levels remain unchanged. (A) Table shows blood serum levels of calcium (Ca), phosphate (Pi) and alkaline phosphatase from 12 week-old FF and KO mice. Data show no differences between FF and KO, either in fed (p>0,05) or fasted state (p>0,05) (n = 13–16). (B) Urine levels of calcium and phosphate are shown relative to creatinine (Cr) excretion levels (n = 9–16). (*p<0,05; **p<0,01). (TIF) [file pone.0102032.s002.tif]

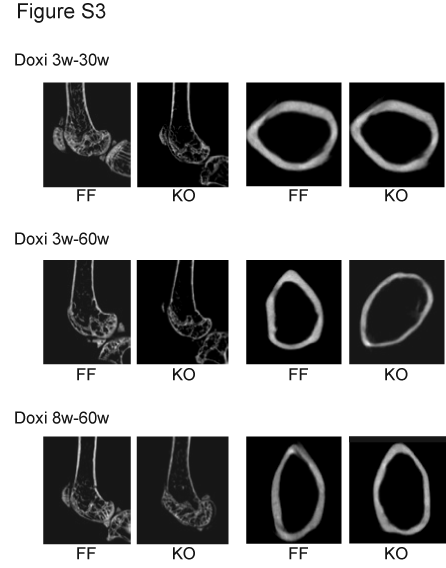

Supplement: Figure S3 — Representative images of distal femur diaphysis and femur cortical bone from control and knockout mice subjected to the distinct doxycycline administration and sacrifice regimes. (TIF) [file pone.0102032.s003.tif]
